# 2020-2021 field seasons of Maize GxE project within the Genomes to Fields Initiative

**DOI:** 10.1186/s13104-023-06430-y

**Published:** 2023-09-14

**Authors:** Dayane Cristina Lima, Alejandro Castro Aviles, Ryan Timothy Alpers, Alden Perkins, Dylan L Schoemaker, Martin Costa, Kathryn J. Michel, Shawn Kaeppler, David Ertl, Maria Cinta Romay, Joseph L. Gage, James Holland, Timothy Beissinger, Martin Bohn, Edward Buckler, Jode Edwards, Sherry Flint-Garcia, Michael A. Gore, Candice N. Hirsch, Joseph E. Knoll, John McKay, Richard Minyo, Seth C. Murray, James Schnable, Rajandeep S. Sekhon, Maninder P. Singh, Erin E. Sparks, Peter Thomison, Addie Thompson, Mitchell Tuinstra, Jason Wallace, Jacob D. Washburn, Teclemariam Weldekidan, Wenwei Xu, Natalia de Leon

**Affiliations:** 1 Department of Agronomy, University of Wisconsin - Madison, Madison, WI 53706 USA; 2 Iowa Corn Promotion Board, Johnston, IA 50131 USA; 3 Institute for Genomic Diversity, Cornell University, Ithaca, NY 14853 USA; 4 Department of Crop and Soil Sciences, North Carolina State University, Raleigh, NC 27695 USA; 5 USDA-ARS Plant Science Research Unit, Raleigh, NC 27606 USA; 6 Department of Crop Science, Center for Integrated Breeding Research, University of Göttingen, Carl-Sprengel-Weg 1, 37075 Göttingen, Germany; 7 University of Illinois at Urbana-Champaign, Urbana, IL 61801 USA; 8 USDA-ARS and Cornell University, Ithaca, NY 14853 USA; 9 USDA ARS CICGRU, 716 Farmhouse Ln, Ames, IA 50011-1051 USA; 10 USDA-ARS, Plant Genetics Research Unit, University of Missouri, 205 Curtis Hall, Columbia, MO 65211 USA; 11 Plant Breeding and Genetics Section, School of Integrative Plant Science, Cornell University, Ithaca, NY 14853 USA; 12 Department of Agronomy and Plant Genetics, University of Minnesota, St Paul, MN 55108 USA; 13 USDA-ARS Crop Genetics and Breeding Research Unit, Tifton, GA 31793 USA; 14 Department of Agricultural Biology, Colorado State University, Fort Collins, CO 80523 USA; 15 Department of Horticulture and Crop Science, College of Food, Agricultural, and Environmental Sciences, Ohio State University, Wooster, OH 44691 USA; 16 Department of Soil and Crop Sciences, Texas A&M University, College Station, TX 77843 USA; 17 Department of Agronomy and Horticulture, University of Nebraska-Lincoln, Lincoln, NE 68588 USA; 18 Department of Genetics and Biochemistry, Clemson University, Clemson, SC 29634 USA; 19 Department of Plant, Soil and Microbial Sciences, Michigan State University, East Lansing, MI 48824 USA; 20 Department of Plant and Soil Sciences, University of Delaware, Newark, DE 19716 USA; 21 Ohio State University, Columbus, OH 43210 USA; 22 Department of Agronomy, Purdue University, West Lafayette, IN 49707 USA; 23 Department of Crop & Soil Sciences, University of Georgia, Athens, GA 30602 USA; 24 Texas A&M University, College Station, TX 77843 USA

**Keywords:** Maize, Genotype by Environment, Grain Yield, Stability, Prediction

## Abstract

**Objectives:**

This release note describes the Maize GxE project datasets within the Genomes to Fields (G2F) Initiative. The Maize GxE project aims to understand genotype by environment (GxE) interactions and use the information collected to improve resource allocation efficiency and increase genotype predictability and stability, particularly in scenarios of variable environmental patterns. Hybrids and inbreds are evaluated across multiple environments and phenotypic, genotypic, environmental, and metadata information are made publicly available.

**Data description:**

The datasets include phenotypic data of the hybrids and inbreds evaluated in 30 locations across the US and one location in Germany in 2020 and 2021, soil and climatic measurements and metadata information for all environments (combination of year and location), ReadMe, and description files for each data type. A set of common hybrids is present in each environment to connect with previous evaluations. Each environment had a collaborator responsible for collecting and submitting the data, the GxE coordination team combined all the collected information and removed obvious erroneous data. Collaborators received the combined data to use, verify and declare that the data generated in their own environments was accurate. Combined data is released to the public with minimal filtering to maintain fidelity to the original data.

## Objective

The release of this data provides a unique resource to understand and dissect genotype-by-environment interactions in maize (*Zea mays* subsp. *mays* L.). Collaborators generate phenotypic, environmental, and metadata datasets to support a more comprehensive understanding of the opportunities and challenges associated with maize production in various environments. The Maize GxE project data is made available to the public in its original form, with minimum filtering to remove erroneous data or as specified by collaborators and in the description files. This approach ensures that the publicly available data contains the maximum amount of information collected by project collaborators and empowers users to define their quality controls based on their specific goals.

A set of 1184 publicly available hybrids were evaluated in the 2020 and 2021 seasons across 30 different locations. The main group of hybrids was produced by the cross of doubled-haploid (DH) inbred lines from the Wisconsin Stiff Stalk MAGIC population (WI-SS-MAGIC), crossed with three ex-PVP inbred testers, PHZ51, PHP02, and PHK76 [1]. The WI-SS-MAGIC population involves the inbreds B73, B84, NKH8431, LH145, PHB47, and PHJ40 as parents in the initial crosses, and a detailed description of the population creation and DH production is in Michel et al. (2022) [1]. The testers were selected to allow adaptation of materials to the wide array of maturities sampled across the project. Inbred tester PHZ51 was used in southern locations (DEH1, GAH1, GAH2, IAH1, IAH2, IAH3, IAH4, MOH1, NCH1, NEH1, NEH2, NEH3, NYH3, SCH1, TXH1, TXH2, TXH3, WIH2), PHK76 in the Midwest and intermediate locations (DEH1, IAH1, IAH2, IAH3, IAH4, ILH1, INH1, MOH1, NCH1, NYH3, WIH2), and PHP02 in the northern locations (DEH1, GEH1, IAH2, IAH3, IAH4, MIH1, MNH1, MOH1, NYH2, OHH1, WIH1, WIH2, WIH3). Six locations (mega-locations) had hybrids created using all three testers (DEH1, IAH2, IAH3, IAH4, MOH1, WIH2). Additional smaller-scale experiments were conducted alongside the main experiment for additional phenotyping and/or deployment of novel phenotyping methods and tools across approximately 83% of the locations. These experiments included the external Yellow Stripes (YS), same set of check hybrids know as ‘Yellow Stripe’ used to connect location and years, but evaluated in a different experiment; the High-Intensity Phenotyping Site (HIPS), which tested 22 hybrids (HIP_Hybrid) and 22 inbreds (HIP_Inbred). HIPS was introduced in 2020 as a more comprehensive phenotyping set for aerial high-throughput phenotyping platforms. The choice of hybrids and inbreds was based on their historical importance and relevance to other connected projects.

## Data description

The 2020 and 2021 datasets are publicly available via CyVerse/iPlant. These datasets contain phenotypic, environmental, soil, and supplemental data, and have been structured according to the specifications outlined in Table 1.

- **Phenotypic data**: Phenotypic measurements that follow a standard set of instructions, available at genomes2fields.org. Standard traits include days to anthesis, days to silking, ear height, plant height, stand count, stalk lodging, root lodging, grain moisture, test weight, plot weight, and grain yield. Both raw data and minimally quality-controlled (clean) data are reported separately. Out of range observations were set to missing values following the rules described in the readMe files.
- **Environmental dataset**: WatchDog 2700 weather stations (Spectrum Technologies) were placed at each field site. Data were collected at 30-min intervals, or according with collaborator set up, from planting through harvest at each location. The geographic locations of the experiments are not identical across years due to crop rotation management practices; thus, the locations of the weather stations vary across years. Each station measured wind speed, direction, and gust; air temperature, dewpoint, relative humidity; soil temperature and moisture; rainfall and solar radiation. Additional measurements taken at selected sites included soil electrical conductivity, ultra-violet light, carbon dioxide, and photosynthetically active radiation. Instructions for weather station maintenance activities including pre-season tasks, field setup, maintenance throughout the growing season, and removal are available on the G2F webpage [2].
- **Soil dataset**: Each field location collected soil samples that represent the experiment field according to the instructions available on the G2F webpage.
- **Supplemental dataset**: Supplemental information consists of metadata (any field-level data collected at planting, in season, and/or at harvest), agronomic information (list of products, nutrients, and irrigation applied), and cooperator list (collaborators responsible for the field locations in 2020 and 2021).

**Table 1:** Overview of 2020 and 2021 planting seasons datasets

Label	Name of data file/data set	File types (Extension)	Data repository and identifier
Data file 1	readme.txt	.txt	CyVerse (10.25739/hzzs-a865) [3]
Data file 2	g2f_2020_phenotypic_data_description.pdf	.pdf	CyVerse (10.25739/hzzs-a865) [3]
Data file 3	g2f_2020_phenotypic_raw_data.csv	.csv	CyVerse (10.25739/hzzs-a865) [3]
Data file 4	g2f_2020_phenotypic_clean_data.csv	.csv	CyVerse (10.25739/hzzs-a865) [3]
Data file 5	g2f_2020_weather_data_description.pdf	.pdf	CyVerse (10.25739/hzzs-a865) [3]
Data file 6	g2f_2020_weather_readMe.txt	.txt	CyVerse (10.25739/hzzs-a865) [3]
Data file 7	2020_weather_raw.csv	.csv	CyVerse (10.25739/hzzs-a865) [3]
Data file 8	2020_weather_cleaned.csv	.csv	CyVerse (10.25739/hzzs-a865) [3]
Data file 9	g2f_2020_soil_data_description.pdf	.pdf	CyVerse (10.25739/hzzs-a865) [3]
Data file 10	g2f_2020_soil_data.csv	.csv	CyVerse (10.25739/hzzs-a865) [3]
Data file 11	g2f_2020_supplemental_information.pdf	.pdf	CyVerse (10.25739/hzzs-a865) [3]
Data file 12	g2f_2020_agronomic_information.csv	.csv	CyVerse (10.25739/hzzs-a865) [3]
Data file 13	g2f_2020_cooperators_list.csv	.csv	CyVerse (10.25739/hzzs-a865) [3]
Data file 14	g2f_2020_field_metadata.csv	.csv	CyVerse (10.25739/hzzs-a865) [3]
Data file 15	readme.txt	.txt	CyVerse (10.25739/5ae3-sw62) [4]
Data file 16	g2f_2021_data_description.pdf	.pdf	CyVerse (10.25739/5ae3-sw62) [4]
Data file 17	g2f_2021_phenotypic_raw_data.csv	.csv	CyVerse (10.25739/5ae3-sw62) [4]
Data file 18	g2f_2021_phenotypic_clean_data.csv	.csv	CyVerse (10.25739/5ae3-sw62) [4]
Data file 19	g2f_2021_weather_data_description.pdf	.pdf	CyVerse (10.25739/5ae3-sw62) [4]
Data file 20	g2f_2021_weather_readMe.txt	.txt	CyVerse (10.25739/5ae3-sw62) [4]
Data file 21	g2f_2021_weather_raw.csv	.csv	CyVerse (10.25739/5ae3-sw62) [4]
Data file 22	g2f_2021_weather_cleaned.csv	.csv	CyVerse (10.25739/5ae3-sw62) [4]
Data file 23	g2f_2021_soil_data_description.pdf	.pdf	CyVerse (10.25739/5ae3-sw62) [4]
Data file 24	g2f_2021_soil_data.csv	.csv	CyVerse (10.25739/5ae3-sw62) [4]
Data file 25	g2f_2021_supplemental_information.pdf	.pdf	CyVerse (10.25739/5ae3-sw62) [4]
Data file 26	g2f_2021_agronomic_information.csv	.csv	CyVerse (10.25739/5ae3-sw62) [4]
Data file 27	g2f_2021_cooperators_list.csv	.csv	CyVerse (10.25739/5ae3-sw62) [4]
Data file 28	g2f_2021_field_metadata.csv	.csv	CyVerse (10.25739/5ae3-sw62) [4]

## Limitations

These datasets contain missing data. Missing data includes data not reported by collaborators or erroneous data as specified on the readMe and description files. Genotypic data is not included in this release. Locations that did not collect data due to the complete loss of the experiment are listed on the cooperator list.

## Data Availability

The data described in this Data note can be freely and openly accessed on CyVerse at 10.25739/hzzs-a865 (2020 Field Season [3]) and 10.25739/5ae3-sw62 (2021 Field Season [4]).

## Abbreviations

G2F: Genomes to Fields
DH: doubled-haploid
GxE: Genotype by environment
